# The effect of health visitors on breastfeeding in Glasgow

**DOI:** 10.1186/1746-4358-1-11

**Published:** 2006-07-05

**Authors:** David Tappin, Jane Britten, Mary Broadfoot, Rhona McInnes

**Affiliations:** 1 Paediatric Epidemiology and Community Health (PEACH) Unit, Department of Child Health, Royal Hospital for Sick Children, Glasgow, UK

## Abstract

**Background:**

The UNICEF Baby Friendly Initiative includes a community component to help women who want to breastfeed. This study aimed to document the health visitor role in promoting and supporting breastfeeding in Glasgow during 2000 and the effect it had on breastfeeding rates.

**Methods:**

Glasgow, UK, has a population of 906,000, with approximately 10,000 births per year. Glasgow has high levels of material deprivation and traditionally low breastfeeding rates. This was a cross-sectional study in January 2000 which used a postal questionnaire to document individual health visitors' interventions, activities and attitude towards breastfeeding. Infant's breastfeeding data collected routinely by the Child Health Surveillance programme from 1 August 1998 to 28 February 1999 was directly matched with interventions, activities and attitudes reported by their own health visitor.

**Results:**

146/216 (68%) health visitors completed and returned the questionnaire. 5401 child health records were eligible and 3,294 (58.2%) could be matched with health visitors who returned questionnaires. 2145 infants had the first visit from 8 to 20 days of age and the second 3 to 7 weeks later. At the first postnatal visit 835 of 2145 (39%) infants were breastfed (median age of 13 days) and 646 (30%) continued to breastfeed at the second visit (median age 35 days).

Infants being breastfed at the first visit were significantly more likely to be fed infant formula at the second visit if their health visitors had had no breastfeeding training in the previous two years (OR1.74 95%CI 1.13, 2.68).

**Conclusion:**

It is essential that Health Visitors are specially trained to support breastfeeding postnatally.

## Background

There is widespread agreement that breastfeeding has health benefits for both babies and mothers, and government health departments are keen to increase breastfeeding rates [1,2]. Recent systematic review of randomised controlled trials [3] concludes that 'supplementary breastfeeding support should be provided as part of routine health service provision and that further trials are required to assess the effectiveness of both lay and professional support in particular in those communities with low rates of breastfeeding initiation. Research is also required into the most appropriate training for those who support breastfeeding mothers.' Evidence exists that the UNICEF Baby Friendly Hospital initiative improves breastfeeding rates [4,5]. Description of what support and who should provide it after leaving hospital is not well defined.

Health visitors have had a role in promoting and supporting breastfeeding for many years [6] but there is little evaluation of their impact on intention and duration [7]. The introduction of the Baby Friendly Initiative award for community health providers [8] based on 'The Seven Point Plan for the Protection, Promotion and Support of Breastfeeding in Community Health Care Settings' has increased interest in breastfeeding work in primary care (Table 1).

**Table 1:** The Seven Point Plan for the Protection, Promotion and Support of Breastfeeding in Community Health Care Settings

**All providers of community health care should:**	
1.	Have a written breastfeeding policy that is routinely communicated to all healthcare staff.
2.	Train all staff involved in the care of mothers and babies in the skills necessary to implement the policy.
3.	Inform all pregnant women about the benefits and management of breastfeeding.
4.	Support mothers to initiate and maintain breastfeeding.
5.	Encourage exclusive and continued breastfeeding, with appropriately-timed introduction of complementary foods.
6.	Provide a welcoming atmosphere for breastfeeding families.
7.	Promote co-operation between healthcare staff, breastfeeding support groups and the local community.

UNICEF UK [8]

The purpose of this study was to document the roles of individual health visitors in promoting and supporting breastfeeding in the primary care setting in Glasgow and to report any relationships between these interventions and routine breastfeeding rates gathered on Child Health Surveillance records linked to individual health visitors.

In the UK, health visitors normally have direct involvement with mothers and babies from around ten days of age when care is passed to them from community midwifery services. Therefore variation in the fall off in breastfeeding rates between the routine Child Health Surveillance data collection points of ten days and six weeks may be an appropriate measure of the effectiveness of breastfeeding support provided by health visitors after controlling for socio-economic factors known to impact on breastfeeding rates [9].

## Methods

The study took place in the year 2000 in the Greater Glasgow Health Board (GGHB) area, with a population of 906,000 and a breastfeeding rate at that time of 37% at 7 days postnatal age [10]. Glasgow with 20% of Scotland's overall population has 80% of those residing in the most materially deprived deprivation category 7 [11]. Within Glasgow City, (population 611,440), 49% of the population live in social rented housing, including large numbers in housing schemes on the edge of the city. There is a small minority ethnic population accounting for some 3.5% of the city's population.

**Figure 1:**
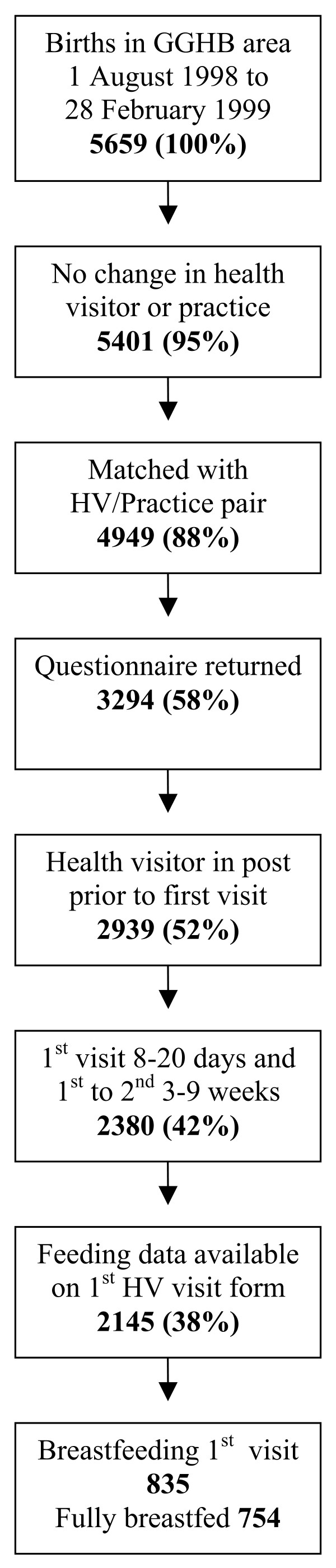
Child Health Surveillance Programme records used.

Infant feeding data and population demographics were collected through the Child Health Surveillance Programme (CHSP) and supplied to us by the Information and Statistics Division, National Health Service (NHS) Scotland. This information is routinely collected for each child at ten days (first health visitor visit), at six weeks (second examination), and at eight months (third examination). CHSP records include identification of GP practice and health visitor enabling them to be linked to health visitor questionnaire data. There were 5659 births in the GGHB area during the period 1 August 1998 to 28 February 1999. CHSP records from 4949 (88%) infants were matched to a GP/HV practice pair, and 3294 (58%) of these records were then matched with a returned questionnaire (Figure 1). CHSP records eligible for inclusion were those where the birth and three CHSP examinations had taken place in the GGHB area and had had the same health visitor and GP at each of the three examinations. A further 1149 CHSP records were excluded from the analysis because the health visitor had not been in post before the 1^st ^CHSP visit (355 records), or the first visit was not between 8 and 20 days after birth or the time between the first and second visit was not between 3 and 9 weeks (559 records), and finally 235 records had no information on breastfeeding at first visit – leaving 2145 (38%) of all birth records (Figure 1). CHSP first health visitor visit records showed 835/2145 (38.9%) infants were breastfed at ten days. These records were used to assess the effect of postnatal intervention described by health visitors on the survival of breastfeeding to the second Child Health Surveillance check scheduled at six weeks.

Health visitor survey data were collected by a postal questionnaire designed specifically for the study. The questionnaire was developed following discussions with practitioners, managers and researchers, and piloted to ensure that it was clear, easy to complete and that the findings would be comprehensive, reliable and valid. The questionnaire collected information about the health visitor's role in supporting breastfeeding: antenatal contact to discuss or provide literature about breastfeeding; postnatal contact including contact with breastfeeding mothers; lactation histories and observed feeds during the week of the 17^th ^to the 21^st ^January 2000; breastfeeding support groups in their GP practice; breastfeeding facilities at their health centre; written breastfeeding policies in their practice or local health care cooperative; written breastfeeding protocol for home visits to breastfeeding mothers; Certificate of Commitment for the Baby Friendly Community Initiative; collection of information on breastfeeding rates; either health visitor or other team members received breastfeeding training in the last two years; other local resources such as breastfeeding volunteers; special funding for breastfeeding work; special responsibilities such as breastfeeding trainer, mentor, helper in a support group; and Health Visitor attitude towards feeding measured by the Iowa Infant Feeding Attitude Scale (IIFAS) [12]. The IIFAS consists of 17 attitude questions, and Health Visitors were asked to respond to each item using a bi-polar 5-point scale (strongly disagree, strongly agree). Approximately one-half of the items were worded in a manner favourable to breastfeeding, and the remaining favourable to formula feeding. Items that favoured formula feeding were reverse scored (i.e., 1 = 5, 2 = 4, 4 = 2, and 5 = 1), and a total attitude score was computed by means of an equally weighted sum of responses to the individual items.

The questionnaire was sent to 229 health visitors in a permanent post with the Greater Glasgow NHS Primary Care Trust on 24 January 2000 to be returned by 3 March 2000 in order to be eligible for a prize draw. All those who returned completed questionnaires were entered in the prize draw and the first 4 names received a €50 John Lewis voucher. A total of 146 health visitor questionnaires were returned in full, giving a crude return rate of 64%, or 68%, following correction for long-term absence (146 of 216). Respondents had been in their present post for between 2 weeks and 28 years, with a median of 5 years. To assess whether those responding were representative of Glasgow health visitors, the caseloads of health visitors who returned questionnaires were compared with those who did not, using data from the Child Health Surveillance programme (Table 2).

**Table 2:** Characteristics from Child Health surveillance records of infants linked to health visitors

**CHS Visit**	**Characteristics**		**Records linked to one HV/GP pair (n1 = 5401)**	**Questionnaire returned (n2 = 3294)**	**Dataset analysed (n3 = 2145)**	p (*)
	Mother's age – years	(mean)	28.4	28.6	28.8	0.04^a^
	Mother employed	(%)	56.2	58.2	61.4	<0.001^b^
	Mother smoker	(%)	33.9	34.3	33.7	NS^b^
	Father's age – years	(mean)	30.9	31.0	31.0	NS^a^
	Father employed	(%)	82.0	82.9	83.8	NS^b^
	Father smoker	(%)	43.0	41.7	42.4	NS^b^
	DEPCAT	(mean)	5.0	4.9	4.9	0.04^a^
						
1	Age – days	(mean)	13.2	13.1	13.2	NS^a^
	Any breastfeeding	(%)	37.8	38.9	38.9	NS^b^
	Only breastfeeding	(%)	33.5	34.7	35.2	NS^b^
						
2	Age – days	(mean)	50.3	50.3	50.3	NS^a^
	Any breastfeeding	(%)	31.4	32.0	32.0	NS^b^
	Only breastfeeding	(%)	23.1	23.5	24.1	NS^b^
						
3	Age – days	(mean)	254	253	252	NS^a^
	Ever breastfed	(%)	45.7	46.7	45.8	NS^b^
	Breastfeeding now	(%)	20.2	20.4	20.6	NS^b^
	Age stopped – weeks	(mean)	3.4	3.5	3.6	NS^a^

* Difference between column n1 and column n3

a t-test for significance of difference of means

b Chi-squared test for significance of difference in proportions

DEPCAT measures material deprivation using small area census data which correlate closely with morbidity and mortality data [11].

DEPCAT 1 = most affluent postcode sectors, DEPCAT 7 = most deprived postcode sectors.

CHS is Child Health Surveillance Programme which includes standard visits to all infants throughout the UK [20].

The statistics package SPSS for Windows was used to perform t-tests to compare mean breastfeeding rates, and logistic regression to detect association between breastfeeding and health visitor interventions, adjusting for Carstairs deprivation category, mother's age, mother in employment and mother smoking. Internal validity of the Iowa Infant Feeding Attitude Scale (IIFAS) was calculated using the Cronbach's alpha statistic in SPSS.

Ethics approval was sought from the Greater Glasgow Community/Primary Care Research Ethics Committee in 1999. The application was examined by the chairman and another member of the committee who replied by letter that no ethical issues were raised by the study and that it could proceed.

## Results

Table 2 shows that infants linked to health visitors (HV) (n1 = 5401), those with returned questionnaires (n2 = 3294) and those analysed (n3 = 2145) had similar socioeconomic characteristics with mothers being slightly older, more likely to be employed and slightly less deprived in the analysed group, but with all datasets having very similar breastfeeding rates.

Table 3 describes the antenatal and postnatal interventions of the 146 HVs who returned their questionnaire. Health visitors were unlikely to have observed an episode of breastfeeding in the designated week (median = 0). There was a high use of materials provided by infant formula manufacturers (73%); 53% used manufacturer-provided weight conversion charts.

**Table 3:** Interventions documented by health visitors to support breastfeeding

**Topics of questions**	**Health visitor responses**	**Number (%) (n = 146)**
Antenatal discussion on benefits & management of breastfeeding	None	48 (33%)
	Leaflets only	73 (50%)
	Discuss with pregnant women	22 (15%)
	Not known	3 (2%)
		
Postnatal contact with breastfeeding mothers	Some contact from 10 days to 6 weeks	45 (31%)
	Weekly contact phone/clinic/etc	38 (26%)
	Weekly home visits	30 (21%)
	Available if mother phones	20 (14%)
	Variable depends on need	12 (8%)
	Not known	1 (1%)
		Median (range)
Contact with breastfeeding mothers (17–21 January 2000)	Breastfeeding mothers in caseload	5 (0–54)
	Mothers initiated contact with each HV	3 (0–50)
	Mothers contacted by each HV	1 (0–20)
	Lactation histories taken by each HV	0 (0–10)
	Breastfeeds observed by each HV	0 (0–10)
		
Health Visitors' use of materials provided by manufacturers of breast milk substitutes	Any use	107 (73%)
	Leaflets/posters on milk feeding/weaning	54 (37%)
	Leaflets/posters on other child care issues	66 (45%)
	Weight conversion charts	78 (53%)
	Obstetric calendars	58 (40%)
	Other calendars	53 (36%)
	Diaries/Diary covers	40 (27%)
	Other	22 (15%)
		
Breastfeeding support groups	None available for HV caseload	40 (27%)
		
Breastfeeding in waiting area at GP surgery where HV practices	All staff would respond positively	63 (43%)
	Private room available if required	57 (39%)
		
Practice has policy/standard or Baby Friendly Certificate of Commitment		7 (5%)
Local Health Care Cooperative has a policy on breastfeeding		21 (14%)
		
Breastfeeding data recorded in HV practice	Initiation of breastfeeding	66 (45%)
	Breastfeeding duration	47 (32%)
		
Breastfeeding training in HVs practice in last 2 years	1. HV received any training	92 (63%)
	2. Course lasting at least 2 days	80 (55%)
	3. Other practice staff received training	15 (10%)
	4. GPs received training	4 (3%)

Table 4 lists the training received by 146 health visitors. Ninety-two (63 per cent) reported receiving breastfeeding training in 1998 or 1999. Of these, 80 (55 per cent) had attended a course lasting two days or more. Eight (6 per cent) had attended a course lasting 2 days or more in both years. Only 14 health visitors, 10 per cent of all respondents reported that other staff in their practice(s) had received any breastfeeding training. Five (3 per cent) reported GPs attending a breastfeeding seminar and 9 (6 per cent) reported training for staff nurses, practice nurses, district nurses or receptionists.

**Table 4:** Breastfeeding training attended by health visitors

**Type of training**	**1998 and 1999 n = 146 (%)**
Lactation Management course, based on UNICEF UK Baby Friendly Initiative Training – 2 days plus mentoring	53 (37)
Training as Trainer or Mentor	6 (4)
Annual 1/2 day breastfeeding update for HVs in Maternity catchment area	5 (3)
BEST workshops: training in association with Breastfeeding Volunteers Initiative	21 (14)
Other training	7 (5)
Total	92 (63)

Table 5 describes their Iowa Infant Feeding Attitude Scale (IIFAS) scores. The possible range is 17 (pro-infant formula feeding) to 85 (pro-breastfeeding). A Cronbach alpha score of 0.79 indicates good internal validity of the IIFAS rating scale. The median score was 72 with a large range from 28 to 85 indicating that some Health Visitors had an attitude that was generally pro-infant formula feeding while most were generally pro-breastfeeding. There was no significant association between quartile of attitude score and health visitor training in the last two years (p = 0.63). The 2145 CHSP first visit infant records which could be matched with health visitors who had returned a questionnaire, showed that 835/2145 (38.9%) infants were being breastfed and 754 (35.2%) were receiving breast milk only.

**Table 5:** Attitudes of 146 health visitors from the Iowa Infant Feeding Attitude Scale

	**SD**-strongly disagree, **D**-disagree, **N**-neutral, **A**-agree, **SA**-strongly agree	**SD **%	**D **%	**N **%	**A **%	**SA **%
1.	The benefits of breast milk last only as long as the baby is breast fed*	76	11	6	3	3
2.	Formula feeding is more convenient than breastfeeding*	56	24	12	3	5
3.	Breastfeeding increases mother infant bonding	3	6	16	20	55
4.	Breast milk is lacking in iron*	40	26	18	6	9
5.	Formula fed babies are more likely to be overfed than breastfed babies	3	11	20	37	30
6.	Formula feeding is the better choice if the mother plans to go back to work*	39	39	19	3	1
7.	Mothers who formula feed miss one of the great joys of motherhood	12	12	28	24	24
8.	Women should not breastfeed in public places such as restaurants*	82	10	3	2	3
9.	Breastfed babies are healthier than formula fed babies	3	5	16	32	45
10.	Breastfed babies are more likely to be overfed than formula fed babies*	60	21	14	2	3
11.	Fathers feel left out if a mother breast feeds*	16	23	42	18	1
12.	Breast milk is the ideal food for babies	7	0	0	2	91
13.	Breast milk is more easily digested than formula	6	2	0	8	84
14.	Formula is as healthy for an infant as breast milk*	44	29	19	6	2
15.	Breastfeeding is more convenient than formula	3	2	12	29	54
16.	Breast milk is cheaper than formula	5	3	5	10	77
17.	A mother who occasionally drinks alcohol should not breastfeed her baby*	47	31	15	3	4

*Variables reverse scored to calculate total infant feeding attitude so that a strongly breastfeeding attitude has a score of 5 for each question giving a maximum score of 85 and minimum of 17

Mean score 71.2 SD 8.4 Cronbach's a = 0.79, median 72, range 28–85, interquartile range 66–77

Table 6 shows the strong association between socioeconomic variables reported on the Child Health Surveillance record and type of infant feeding at first HV visit. Infants of young mothers were far more likely to be fed infant formula as were infants of mothers who smoked, were unemployed or who lived in areas of material deprivation. Of infants who were breastfed at their first postnatal visit 825/835 had information recorded about feeding at their second visit and 179/825 (21.7%) had stopped breastfeeding in the 3–9 weeks between these visits.

**Table 6:** Univariate and Multivariate analysis describing the association between socio-economic factors and infant formula feeding at 1^st ^visit (n = 2145)

**Maternal status**	**No of infants**	**Infants fed formula (%)**	**OR (95%CI)**	**p**	Adjusted OR* (95%CI)	**p**
**Age in years**						
> 34	340	183 (54)	1		1	
30–34	647	321 (50)	0.85 (0.65,1.10)	0.21	0.84 (0.60,1.17)	0.30
25–29	524	319 (61)	1.34 (1.01,1.76)	0.04	1.06 (0.75,1.50)	0.73
20–24	312	244 (78)	3.08 (2.18,4.34)	<0.001	1.83 (1.20,2.82)	<0.01
<20	179	158 (88)	6.46 (3.91,10.67)	<0.001	6.24 (2.84,13.67)	<0.001
Not Known	143					
**Mother Smoker**						
No	1167	615 (53)	1		1	
Yes	593	482 (81)	3.89 (3.07,4.93)	<0.001	2.44 (1.84,3.22)	<0.001
Not Known	385					
**Mother employed**						
Yes	1035	542 (52)	1		1	
No	649	467 (72)	2.33 (1.89,2.88)	<0.001	1.39 (1.08,1.78)	<0.01
Not Known	461					
DEPCAT **						
1	193	55 (29)	1		1	
2	165	66 (40)	1.67 (1.08,2.60)	0.02	0.90 (0.51,1.58)	0.71
3	222	124 (56)	3.18 (2.11,4.78)	<0.001	2.81 (1.69,4.69)	<0.001
4	218	108 (50)	2.46 (1.64,3.71)	<0.001	1.82 (1.10,3.02)	0.02
5	254	159 (63)	4.20 (2.81,6.28)	<0.001	3.02 (1.82,5.03)	<0.001
6	462	288 (62)	4.15 (2.88,5.98)	<0.001	2.38 (1.50,3.78)	<0.001
7	600	490 (82)	11.18 (7.68,16.26)	<0.001	5.49 (3.42,8.84)	<0.001
Not Known	31					

* Adjustment was made for the other three socioeconomic variables in the table

** DEPCAT 7-most deprived, 1-most affluent.

Table 7 presents the univariate and multivariate analysis of the association between Health Visitor related factors and continued breastfeeding at second visit. Infants looked after by health visitors who had had no breastfeeding training in the previous two years were nearly twice as likely to stop breastfeeding compared with infants looked after by health visitors who had had training. Weekly visits as a routine were associated with an increase in the proportion who stopped breastfeeding compared with contact initiated by the health visitor at least once during the first 6 weeks (p = 0.03). This association may have been confounded, for instance by Health Visitor experience if new health visitors were more likely to choose a fixed weekly contact compared to more experienced practitioners. The association became non-significant (p = 0.07) when training in the previous two years was taken into account. A weak association was seen between HV attitude and continued breastfeeding which did not reach statistical significance. Availability of breastfeeding support groups and use of commercial company products by HVs had little effect on breastfeeding survival at second visit.

**Table 7:** Univariate and Multivariate analysis describing the association between Health Visitor related factors and continued breastfeeding at 2^nd ^visit for those who were breastfeeding at first visit (n = 825)

	**Infants breastfed at 1^st ^visit**	**Breastfeeding stopped by 2^nd ^visit (%)**	**OR (95%CI)**	**p**	Adjusted OR* (95%CI)	**p**
**HV breastfeeding support training 1998 or 1999**						
Yes	549	105 (19)	1		1	
No	263	71 (27)	1.56 (1.11,2.21)	0.01	1.74 (1.13,2.68)	0.01
Not Known	13					
						
**Postnatal contact practiced by HV**						
Weekly visits	151	44 (29)	1		1	
Contact from HV	412	79 (19)	0.58 (0.38,0.89)	0.01	0.55 (0.32,0.94)	0.03
Available if called	194	44 (23)	0.71 (0.44,1.16)	0.17	0.73 (0.39,1.36)	0.32
Not Known	68					
						
**Feeding attitude of health visitors (IIFAS)**						
*Pro-breast feeding*						
Highest quartile	207	38 (18)	1		1	
Quartile 2	206	45 (22)	1.22 (0.77,2.02)	0.38	1.30 (0.71,2.38)	0.39
Quartile 3	206	48 (23)	1.35 (0.84,2.17)	0.22	1.57 (0.88,2.84)	0.13
Lowest quartile	206	48 (23)	1.35 (0.84,2.17)	0.22	1.25 (0.68,2.31)	0.48
*Pro-infant formula feeding*						
						
**HV uses commercial company leaflets**						
No	187	45 (19)	1		1	
Yes	445	132 (23)	1.23 (0.84,1.80)	0.28	1.19 (0.74,1.91)	0.48
Not Known	193					
						
**Breastfeeding support groups available to mothers**						
Yes	597	125 (21)	1		1	
No	228	54 (24)	1.17 (0.81,1.69)	0.39	1.40 (0.87,2.22)	0.16

* Adjustment was made for maternal age, smoking status, employment status and DEPCAT

## Discussion

This observational study attempted to examine if health visitors had an effect on the initiation and survival of breastfeeding from first visit at 10 days postnatal age to second visit at 6 weeks postnatal age for infants born from 1 August 1998 to 28 February 1999 in Glasgow, an area of severe material deprivation. Outcome data on breastfeeding was collected routinely via the Child Health Surveillance system. Health visitor questionnaire was completed after breastfeeding data collection so would not have changed the outcome. The main finding was that infants who were breastfed at the first routine health visitor contact after birth were nearly twice as likely to continue to be breastfeeding at the second routine contact if their health visitor had received training in breastfeeding support in the previous two years (Table 7, OR 1.74). This association remained significant after controlling for socioeconomic variables collected using logistic regression analysis. This finding is important and should encourage health service managers to ensure that health professionals who come into contact with breastfeeding mothers receive adequate training. An appropriate evidence based course for health professionals may be the Baby Friendly Initiative's three day course in breastfeeding management which is designed to provide health professionals with the practical skills they need to successfully implement best practice standards [13]. There is some evidence to suggest that Baby Friendly Initiative training may be helpful in improving attitudes, knowledge and skills of health visitors, but evidence is from methodologically poor studies [[14], p 125][15]. Further work is needed to look at the effectiveness of different training packages for health visitors and their impact on breastfeeding rates.

This observational study of routine practice was designed to examine the health visitors' role in supporting breastfeeding in Glasgow, one of the most deprived cities in Europe, and the effect interventions had on breastfeeding rates of a cohort of infants born from 1 August 1998 to 28 February 1999. It has the advantage of large size within a real life setting and the disadvantage of possible unknown confounding factors being responsible for associations described. Although pragmatic trials are often used, randomised controlled trials tend to have well trained practitioners who have positive attitudes towards breastfeeding. This observational study describes health visitors in their normal working environment where not everybody is well trained as shown in Table 4 and not everybody has a positive attitude towards breastfeeding as shown in Table 5.

The Iowa Infant Feeding Attitude Scale (IIFAS) has previously been tested for reliability and validity in a series of studies of women in the USA [12], and among fathers and mothers of infants in Glasgow [16,17]. The IIFAS was found in these studies to have good internal consistency, with a Cronbach's alpha of 0.79 and 0.77 for mothers and fathers respectively in Glasgow [16,17]. Higher scores predicted subsequent breastfeeding. The present study is the first time the IIFAS scale has been used to describe feeding attitudes of health care workers. Most health visitors had a positive attitude towards breastfeeding but 25% had a level of 66 and below. Breastfeeding mothers at discharge in Glasgow had a mean score of 65 in 2000 [16]. This would suggest that 25% of health visitors who responded to the questionnaire had a more negative attitude towards breastfeeding than perhaps half the breastfeeding women in Glasgow. Many of the 36% of health visitors who did not respond to the questionnaire may also have been in this category.

The drawback of this situation has been highlighted by our study which suggests that interventions may not be risk-free (regular weekly visits compared with contact from the health visitor as she feels is required – see Table 7) if attempted by health visitors without adequate training and with perhaps attitudes favouring infant formula. There is limited evidence from other UK studies about the impact of health professional breastfeeding training on duration of breastfeeding, with only one study including health visitors [7,14,15,18].

It should be highlighted that 73% of health visitors (Table 3) used materials provided by manufacturers of breast milk substitutes. This is contrary to the World Health Organization Code: – International Code of Marketing of Breast-milk substitutes [19]. Article 6.2 states "No facility of a health care system should be used for the purposes of promoting infant formula or other products within the scope of this Code".

## Conclusion

This study complements the randomised controlled trials of postnatal support for breastfeeding by emphasising the need for health visitors in the UK to be specially trained to provide support for breastfeeding mothers.

## Competing interests

The author(s) declare that they have no competing interests.

## Authors' contributions

DT applied for funding, designed, supervised and helped analyse the data. He also wrote the final article.

JB helped design the study, performed data collection and collation and helped write the final article.

MB helped design the study, performed data collection and collation and analysed the data as well as helping write early drafts of the article.

RM wrote an earlier draft of the article and gave advice on design.
